# Quality of care and its determinants in longer term mental health facilities across Europe; a cross-sectional analysis

**DOI:** 10.1186/s12888-016-0737-5

**Published:** 2016-02-11

**Authors:** Helen Killaspy, Graça Cardoso, Sarah White, Christine Wright, José Miguel Caldas de Almeida, Penny Turton, Tatiana L. Taylor, Matthias Schützwohl, Mirjam Schuster, Jorge A. Cervilla, Paulette Brangier, Jiri Raboch, Lucie Kalisova, Georgi Onchev, Spiridon Alexiev, Roberto Mezzina, Pina Ridente, Durk Wiersma, Ellen Visser, Andrzej Kiejna, Tomasz Adamowski, Dimitris Ploumpidis, Fragiskos Gonidakis, Michael King

**Affiliations:** Division of Psychiatry, University College London, Maple House, 149 Tottenham Court Road, London, UK; CEDOC and Mental Health Department, Faculdade de Ciências Médicas, Universidade Nova de Lisboa, Campo Mártires da Pátria 130, 1169-056 Lisbon, Portugal; Population Health Research Institute, St George’s University London, Cranmer Terrace, London, UK; Department of Psychiatry and Psychotherapy, University Hospital, Technische Universitaet Dresden, Fetscherstrasse 74, 01307 Dresden, Germany; CIBERSAM, Department of Psychiatry, Universidad de Granada, Avenida de Madrid 11, 18012 Granada, Spain; Department of Psychiatry, lst Faculty of Medicine, Charles University, Ke Karlovu 11, 128 00 Prague 2, Czech Republic; Department of Psychiatry, Medical University Sofia, St Georgi Sofiisky str. L, Sofia, 1431 Bulgaria; Dipartimento di Salute Mentale, Via Sai, 1-3, Trieste, 34127 Italy; University Medical Centre, Hanzeplein1, Groningen, 9700 RB Netherlands; Department of Psychiatry, Wroclaw Medical University, Pasteura 10, Wroclaw, 50-367 Poland; University Mental Health Research Institute, Soranou Tou Efessiou 2, Athens, 11527 Greece

## Abstract

**Background:**

The Quality Indicator for Rehabilitative Care (QuIRC) is an international, standardised quality tool for the evaluation of mental health facilities that provide longer term care. Completed by the service manager, it comprises 145 items that assess seven domains of care: living environment; treatments and interventions; therapeutic environment; self-management and autonomy; social interface; human rights; and recovery based practice. We used the QuIRC to investigate associations between characteristics of longer term mental health facilities across Europe and the quality of care they delivered to service patients.

**Methods:**

QuIRC assessments were completed for 213 longer term mental health units in ten countries that were at various stages of deinstitutionalisation of their mental health services. Associations between QuIRC domain scores and unit descriptive variables were explored using simple and multiple linear regression that took into account clustering at the unit and country level.

**Results:**

We found wide variation in QuIRC domain scores between individual units, but across countries, fewer than a quarter scored below 50 % on any domains. The quality of care was higher in units that were smaller, of mixed sex, that had a defined expected maximum length of stay and in which not all patients were severely disabled.

**Conclusions:**

This is the first time longer term mental health units across a number of European countries have been compared using a standardised measure. Further use of the QuIRC will allow greater understanding of the quality of care in these units across Europe and provide an opportunity to monitor pan-European quality standards of care for this vulnerable patient group.

## Background

The European Commission’s Green Paper on Mental Health in 2005 [1] specifically highlighted the need to improve the social inclusion of mentally ill people and to protect their fundamental rights and dignity. It acknowledged the stigma and prejudice they often face and the evidence for better quality of life and improved social inclusion that has arisen from the deinstitutionalisation of mental health services in many countries. However, it also acknowledged that in some Member States, institutions still account for a large share of the mental health services infrastructure and, in many countries are poorly covered by existing health monitoring systems. It highlighted the need to harmonize indicators and information about these facilities across the EU, such that recommendations for best practice in promotion of social inclusion and the protection of human rights of people with longer term mental health problems could be made. The DEMoBinc study was funded by the European Commission [2] to develop an international toolkit, the Quality Indicator for Rehabilitative Care (QuIRC), to assess the quality of care in psychiatric and social care facilities for adults with longer term mental health problems. It is one facet of the work needed in the EU’s agenda for quality assurance. The development of the QuIRC has been described elsewhere [3].

In summary, evidence from the results of a systematic literature review [4], international Delphi exercise [5] and review of care standards in each of the ten participating countries was triangulated to derive items for the QuIRC. Items were included if there was evidence of their ability to support the recovery of individuals with longer term mental health problems. The QuIRC comprises 145 items of which 86 are used to assess seven domains of care: the Living (built) Environment; the Treatments and Interventions provided, including the use of restraint and seclusion; the Therapeutic Environment (culture of the unit); the promotion of patients’ Self-management and Autonomy; the promotion of their Social Interface with the community and family/carers; the protection of their Human Rights; the implementation of Recovery Based Practice. The remaining items provide descriptive data. The QuIRC is available as a web based application (www.quirc.eu) in the ten languages of the countries that participated in its development. Results are presented in a printable report showing the unit’s performance on each domain as a percentage, on a “spider web” diagram, which also shows the average performance for similar units in the same country. Higher domain scores denote better quality of care in each domain. Although it is known that community based units tend to have a more homely environment [4, 6], whereas inpatient units tend to have a greater proportion of service users with more complex needs who are detained involuntarily, the impact on the quality of care of other characteristics of longer term mental health facilities is not known. Therefore, our aim was to explore associations between the characteristics of units that participated in the DEMoBinc study and the quality of care as assessed by the QuIRC.

## Methods

### Setting

Ten European countries participated in the DEMoBinc study (Bulgaria, Czech Republic, Germany, Greece, Italy, Netherlands, Poland, Portugal, Spain and the UK), purposively selected to represent countries at different stages of deinstitutionalisation and a broad geographical spread across the European Union. Since the aim of the DEMoBinc study was to develop a quality assessment tool that could be used across hospital and community based longer term mental health care facilities in different countries, rather than to carry out a representative survey of such facilities, services meeting specific criteria were purposively identified for participation by the principal investigator in each country from personal knowledge and local registration lists. All had to provide longer term care (length of stay at least 6 months), for at least six service users living in a communal setting (i.e. not individual flats/bedsits), with staff on-site, usually 24 h a day. Units that only provided for specialist groups such as those with learning disability, degenerative brain disease or head injuries, substance misuse or dementia were excluded. Both hospital and community based units of different size and geographical location (urban/suburban/rural) were purposively recruited. Most users of these services were male, with a diagnosis of psychotic illnesses such as schizophrenia and a mean length of stay of 9 months (see Killaspy et al. [7], for more details). Face to face interviews with unit managers were carried out by the researchers in each country in order to complete the QuIRC. Managers were first contacted by the researchers about the study who explained its purpose and process. If they were willing, the researcher arranged to meet with them and they were sent a Participant Information Sheet. They were given at least 2 days to review this and were then able to clarify any queries with the researcher before given informed consent to participate. The study was approved by the relevant ethics committees in each of the ten participating countries involved in developing the QuIRC (Bulgaria - Ethics Committee, Alexandrovska University Hospital, Sofia; Czech Republic - General University Hospital, Ethics Committee, Prague; Germany – Ethik Kommission der Medizinischen Fakultät Carl Gustav Carus an der Technischen Universität, Dresden; Greece - University Medical Mental Health Research Institute, Athens; Italy - Comitato Etico Indipendente, Trieste; the Netherlands - Medical Ethical Committee of the University Medical Centre, Groeningen; Poland - Commission of Bioethics, Wroclaw Medical University; Portugal - Ethical Committee of the New University of Lisbon Medical School; Spain - Comisión Etica de la Universidad de Granada; UK - City and East London Multi Region Ethics Committee).

### Data analysis

Using data from a report by Dunlap, Xin & Myers [8], it was calculated that with a sample size of 213 units there would be 80 % power to estimate the relationship of ten variables of a small to medium effect size of 0.33 with each domain of QuIRC at a 0.7 % significance level. This significance level takes account of the fact that seven regression models were being fitted.

The following unit characteristics were investigated for their association with QuIRC domain scores: unit type (hospital or community based); location (urban, suburban, rural); size (total number of beds); whether there was a maximum length of stay; whether the unit was single or mixed gender; the proportion of patients generally able to do very little without assistance; the proportion of patients detained involuntarily; staffing intensity (ratio of the number of full-time staff to beds); and staff turnover (the proportion of staff who had left, retired, died or been dismissed in the previous two years). Descriptive data on the input from different staff disciplines was also recorded in terms of whether the unit had access to the discipline from outside the unit or whether staff were employed within the unit, but these variables could not be included in the model due to very small numbers of staff from some disciplines in some countries.

The QuIRC domain scores were normally distributed and were each analysed as dependent variables for associations with the variables listed above. Multiple linear regression was used, with categorical independent variables entered as dummy variables. It was important to adjust for clustering of units within countries so parameters of the linear regression were computed using robust clustered standard errors, the country identifier being the cluster variable. Un-standardised regression coefficients and their 95 % confidence intervals (estimated using bootstrapping) are presented for all parameter estimates.

## Results

### Unit characteristics

Table 1 shows the units’ characteristics. The QuIRC was completed in 213 units across 10 European countries. Around half of these units were in the inner city, and two-thirds were community based facilities. The size of units varied greatly (IQR from 12 to 35 patients). The majority (*n* = 172, 81 %) did not have a maximum length of stay, but where present this was usually 2 years. One quarter of units were single sex. The majority of units (59 %) had no detained patients, while in a small number (*n* = 14, 7 %) more than 50 % of patients were detained. The functional impairment of patients varied, with about one quarter of units having no patients who were able to do very little without assistance, but in 23 (8 %) units the majority of patients required assistance with most things.

**Table 1:** Units’ Descriptive statistics, by country, n (%) unless otherwise stated

Variable	Label	Bul	CzR	Ger	Gre	Ita	Neth	Pol	Port	Spa	UK	Total
	Number of units surveyed	20	20	21	22	29	21	20	20	20	20	213
Location	*Inner city*	6 (30 %)	6 (30 %)	11 (52 %)	20 (91 %)	14 (48 %)	4 (19 %)	11 (55 %)	9 (45 %)	13 (65 %)	15 (75 %)	109 (51 %)
	*Suburbs*	5 (25 %0	12 (60 %)	4 (19 %)	0 (0 %)	12 (41 %)	11 (52 %)	9 (45 %)	3 (15 %)	6 (30 %)	5 (25 %)	67 (32 %)
	*Countryside*	9 (45 %)	2 (10 %)	6 (29 %)	2 (9 %)	3 (11 %)	6 (29 %)	0 (0 %)	8 (40 %)	1 (5 %)	0 (0 %)	37 (17 %)
Type	*Community*	12 (60 %)	5 (25 %)	21 (100 %)	22 (100 %)	29 (100 %)	11 (52 %)	10 (50 %)	7 (35 %)	10 (50 %)	15 (75 %)	142 (67 %)
	*Hospital*	8 (40 %)	15 (75 %)	0 (0 %)	0 (0 %)	0 (0 %)	10 (48 %)	10 (50 %	13 (65 %)	10 (50 %)	5 (25 %)	71 (33 %)
Unit size	*Mean (SD)* *Range*	53.8 (33.5) 8 – 120	34.5 (18.4) 5 – 56	23.3 (12.7) 9 – 58	12.9 (3.9) 6 – 20	10.4 (4.6) 5 – 24	30.2 (14.0) 11 – 60	46.3 (13.8) 31 – 73	16.5 (10.5) 6 – 40	21.4 (14.6) 11 - 70	14.1 (5.3) 6 – 30	25.5 (20.1) 5-120
Has max length of stay		1 (5 %)	3 (15 %)	0 (0 %)	5 (23 %)	10 (35 %)	1 (5 %)	0 (0 %)	2 (10 %)	2 (10 %)	17 (85 %)	41 (19 %)
Single gender units	*Male*	7 (35 %)	7 (35 %)	0 (0 %)	1 (5 %)	2 (7 %)	0 (0 %)	5 (25 %)	6 (30 %)	2 (10 %)	1 (5 %)	31 (15 %)
	*Female*	4 (20 %)	5 (25 %)	0 (0 %)	1 (5 %)	2 (7 %)	0 (0 %)	4 (20 %)	2 (10 %)	0 (0 %)	1 (5 %)	19 (9 %)
Detained patients	*% in unit, mean (SD) range*	13.1 (24) 0 – 96	7.9 (13) 0 – 50	3.5 (9) 0 – 29	12.3 (27) 0 – 100	3.2 (7) 0 -30	11.7 (19) 0 – 65	17.6 (25) 0 – 100	0.3 (1) 0 – 5	17.2 (35) 0 – 100	23.4 (25) 0 – 75	10.7 (21) 0 – 100
	*>50 % pts involuntary*	1 (5 %)	0 (0 %)	0 (0 %)	3 (14 %)	0 (0 %)	2 (10 %)	2 (10 %)	0 (0 %)	3 (15 %)	3 (15 %)	14 (6.6 %)
% Pts that need high levels of assistance	*None*	4 (20 %)	6 (30 %)	5 (24 %)	14 (64 %)	11 (38 %)	3 (14 %)	2 (10 %)	5 (25 %)	2 (10 %)	6 (30 %)	58 (27 %)
	*>50 %*	3 (15 %)	1 (5 %)	1 (5 %)	1 (5 %)	1 (3 %)	6 (29 %)	2 (10 %)	3 (15 %)	4 (20 %)	1 (5 %)	23 (8 %)
Staffing	*Intensity (Full Time Equivalent staff to bed ratio), mean (SD) range*	0.2 (0.2) 0.1 – 0.6	0.5 (0.2) 0.1 – 0.8	0.3 (0.2) 0.1 – 0.7	1.7 (1.9) 0.1 – 9.1	0.2 (0.1) 0.1 – 0.5	0.6 (0.4) 0.2 – 1.8)	0.5 (0.2) 0.2 – 0.9	0.3 (0.3) 0.1 – 1.1	1.6 (1.2) 0.3 – 4.7	1.2 (0.5) 0.1 – 1.9	0.7 (0.9) 0.1 – 9.1
	*Turnover (per bed in past 2 years), mean (SD) range*	0.12 (0.13) 0 – 0.55	0.22 (0.25) 0 – 0.86	0.23 (0.31) 0 – 1.00	0.13 (0.13) 0 – 0.44	0.21 (0.16) 0 – 0.75	0.58 (0.90) 0 – 4.00	0.13 (0.12) 0 – 0.37	0.14 (0.17) 0 – 0.50	0.19 (0.33) 0 – 1.50	0.11 (0.09) 0 – 0.30	0.21 (0.36) 0 – 4.0
	*Psychiatrist in the unit*	9 (45 %)	16 (80 %)	0 (0 %)	18 (82 %)	21 (72 %)	12 (57 %)	20 (100 %)	10 (50 %)	14 (70 %)	15 (75 %)	135 (63 %)
	*No Psychiatrist input*	0 (0 %)	1 (5 %)	2 (10 %)	0 (0 %)	1 (3 %)	0 (0 %)	0 (0 %)	0 (0 %)	0 (0 %)	0 (0 %)	4 (2 %)
	*Clinical Psychologist*	4 (20 %)	12 (60 %)	1 (5 %)	17 (77 %)	16 (55 %)	4 (19 %)	19 (95 %)	6 (30 %)	14 (70 %)	12 (60 %)	105 (49 %)
	*Social Worker*	0 (0 %)	20 (100 %)	7 (33 %)	18 (82 %)	23 (79 %)	9 (43 %)	15 (75 %)	14 (70 %)	13 (65 %)	1 (5 %)	120 (56 %)
	*Vocational rehabilitation lead*	14 (70 %)	1 (5 %)	5 (24 %)	14 (64 %)	0 (0 %)	8 (38 %)	0 (0 %)	3 (15 %)	14 (70 %)	10 (50 %)	69 (32 %)

All units employed nurses and support workers. Two-thirds of units employed a psychiatrist, but only half employed a clinical psychologist, only half employed a social worker, and one-third employed a lead for vocational rehabilitation. The mean number of full time equivalent (FTE) staff to beds was 0.4 (4 staff to 10 beds) but this ranged from 0.1 to 9.1 across units. Staff turnover in the last 2 years (per unit bed) ranged from 0.0 to 4.0.

### Associations between unit quality (QuIRC domain scores) and unit characteristics

Table 2 shows mean and standard deviation QuIRC domain scores by country.

**Table 2:** Domain scores by country, mean (SD), minimum - maximum

	Bul	CzR	Ger	Gre	Ita	Neth	Pol	Port	Spa	UK	Overall
Living environment	54.1 (18.1) 32.0 - 82.8	52.4 (15.1) 30.3 - 84.4	73.8 (7.9) 58.2 - 89.3	58.0 (7.6) 46.7 - 71.3	64.8 (9.6) 45.1 - 86.1	70.1 (14.0) 40.2 - 89.3	49.0 (12.9) 32.0 - 71.3	59.2 (15.6) 35.2 - 89.3	46.5 (16.8) 15.6 - 71.3	67.0 (10.7) 48.4 - 86.1	59.8 (15.5) 15.6 - 89.3
Therapeutic Environment	45.6 (12.2) 20.9 - 72.2	51.2 (8.9) 37.1 - 77.2	51.2 (8.9) 37.1 - 77.2	52.1 (8.6) 35.8 - 66.3	52.6 (6.8) 40.8 - 69.2	51.6 (4.9) 41.7 - 59.9	47.5 (8.6) 34.8 - 62.3	47.8 (10.5) 26.8 - 64.2	55.7 (8.0) 43.1 -67.5	64.5 (6.0) 58.1 - 78.4	52.1 (9.5) 21.0 - 78.4
Treatments and Interventions	48.5 (11.4) 29.1 - 69.2	49.3 (9.4) 37.9 - 79.6	51.6 (8.5) 33.7 - 63.1	47.4 (6.4) 33.1 - 58.1	50.6 (6.7) 37.5 - 66.8	52.7 (7.1) 39.5 - 65.2	46.2 (7.7) 34.3 - 63.6	46.5 (10.1) 28.2 - 65.6	54.0 (9.5) 31.4 - 66.0	59.5 (8.0) 45.8 - 77.3	50.6 (9.1) 28.2 - 79.6
Self-management and Autonomy	44.9 (19.2) 16.6 - 79.4	49.3 (15.6) 28.6 - 82.7	71.9 (8.3) 58.1 - 85.7	59.9 (11.2) 27.9 - 73.8	53.2 (9.1) 34.8 - 71.7	66.0 (9.8) 45.8 - 81.3	44.1 (9.6) 28.7 - 63.9	49.6 (16.5) 20.4 - 80.3	46.9 (10.3) 22.5 - 63.3	68.7 (11.0) 44.7 - 86.0	55.5 (15.5) 16.6 - 86.0
Social Interface	45.8 (17.7) 14.2 - 81.7	48.6 (14.0) 29.9 - 88.8	40.3 (11.5) 22.0 - 64.7	47.3 (11.2) 29.6 - 72.5	50.0(11.9) 31.5 - 75.9	47.0 (9.4) 33.4 - 61.3	40.1 (14.0) 19.0 - 67.1	52.0 (19.3) 16.3 - 85.0	59.5 (16.4) 32.4 - 83.0	53.9 (12.7) 34.9 - 76.3	48.5 (14.7) 14.2 - 88.8
Human Rights	52.4 (14.4) 30.1 - 72.9	55.1 (8.8) 45.6 - 81.1	65.7 (5.7) 53.9 - 74.7	52.9 (11.7) 25.2 - 69.5	48.1 (9.6) 31.7 - 69.3	70.8 (6.4) 59.7 - 82.1	53.0 (10.4) 37.6 - 76.8	48.7 (11.8) 30.8 - 71.2	53.7 (9.1) 36.3 - 70.7	69.6 (9.2) 51.1 - 82.8	56.7 (12.7) 25.2 - 82.8
Recovery Based Practice	45.5 (15.9) 22.9 - 79.6	52.5 (12.3) 32.0 - 80.2	62.4 (8.8) 42.7 - 74.8	56.0 (11.7) 28.7 - 69.9	48.4 (8.1) 34.3 - 66.7	51.7 (8.6) 34.5 - 67.8	46.1 (10.3) 27.1 - 69.1	44.2 (13.4) 15.7 - 66.4	55.4 (8.8) 34.3 - 69.4	65.9 (9.7) 49.0 - 81.6	52.7 (12.7) 15.7 - 81.6

Table 3 shows the association of QuIRC domain scores with unit characteristics after adjustment.

**Table 3:** Association of QuIRC scores for individual domains of care with institutional, patient and staff variables

Variable	Label	Living Environment	Therapeutic Environment	Treatments and Interventions	Self-management and Autonomy	Social Interface	Human Rights	Recovery Based Practice
Unit Type	Community	**11.2 (5.4, 17.3)**	**-3.4 (-6.2, -0.5)**	-3.1 (-6.4, 0.2)	5.4 (-0.9, 11.7)	**-7.8 (-13.2, -2.4)**	-1.8 (-8.6, 4.9)	0.2 (-4.5, 4.8)
Unit Location	Suburbs	0.9 (-4.3, 6.1)	-1.2 (-3.2, 0.8)	0.6 (-2.3, 3.5)	0.3 (-4.5, 5.2)	-0.4 (-3.2, 2.3)	0.7 (-4.3, 5.7)	-2.1 (-4.9, 0.6)
	Country	0.7 (-3.5, 5.0)	**-3.3 (-5.6, -1.1)**	-2.1 (-4.4, 0.2)	0.5 (-4.4, 5.3)	-1.9 (-5.8, 2.0)	-2.2 (-7.4, 2.9)	**-3.7 (-7.4, 0.0)**
Size of unit (bed number)		**-0.2 (-0.3, -0.1)**	**-0.1 (-0.2, 0.0)**	0.0 (-0.1, 0.0)	**-0.2 (-0.4, -0.1)**	**-0.2 (-0.4, -0.1)**	0.0 (-0.2, 0.1)	-0.1 (-0.2, 0.0)
Maximum length of stay	Yes	-2.6 (-9.8, 4.5)	**9.0 (5.3, 12.7)**	**6.0 (2.3, 9.6)**	1.4 (-6.2, 9.0)	**6.7 (0.6, 12.8)**	2.4 (-5.3, 10.0)	**5.8 (0.8, 10.9)**
Gender	Single sex	-5.7 (-12.4, 1.0)	-2.0 (-5.7, 1.8)	-2.8 (-6.3, 0.7)	**-8.6 (-16.5, -0.7)**	-0.1 (-3.7, 3.4)	**-7.9 (-15.3, -0.5)**	**-4.8 (-9.5, -0.2)**
% of residents able to do very little without assistance		-0.1 (-0.2, 0.1)	**-0.1 (-0.2, 0.0)**	**-0.1 (-0.2, -0.1)**	**-0.2 (-0.3, -0.1)**	**-0.2 (-0.3, -0.1)**	**-0.1 (-0.2, 0.0)**	**-0.2 (-0.3, -0.1)**
% of residents detained		**-0.1 (-0.1, 0.0)**	0.0 (0.0, 0.0)	0.0 (0.0, 0.1)	**-0.1 (-0.1, 0.0)**	0.0 (-0.1, 0.1)	0.0 (-0.1, 0.0)	0.0 (-0.1, 0.0)
Staff intensity (ratio of number of full time equivalent staff to number of beds)		-2.7 (-8.4, 3.0)	0.9 (-3.6, 5.4)	0.7 (-3.4, 4.9)	-0.4 (-9.1, 8.2)	1.5 (-2.5, 5.5)	-0.2 (-10.6, 10.1)	1.5 (-5.2, 8.1)
Staff turnover		3.0 (-8.4, 14.4)	1.5 (-0.7, 3.7)	1.3 (-1.6, 4.2)	3.3 (-5.5, 12.0)	2.3 (-1.5, 6.1)	2.4 (-4.0, 8.8)	1.7 (-1.2, 4.6)

Values are unstandardized regression coefficients, (95 % CI). Cells in bold indicate variable is significant at the 5 % level

### Living environment

The Living Environment domain had an overall mean score across countries of 59.8 %, (mean range 73.8 % [Germany] to 46.5 % [Spain]). This was associated with the type of unit, size of unit and the percentage of patients involuntarily detained: community units scored 11.2 % more than hospital units; larger units scored lower on Living Environment, every extra bed reducing the score in this domain by 0.2 %; for every 1 % increase in detained patients the Living Environment score reduced by 0.1 %.

### Therapeutic environment

The Therapeutic Environment domain had an overall mean score across countries of 52.1 % (mean range 64.5 % [UK] to 45.6 % [Bulgaria]). This was associated with the type of unit, location of unit, size of unit, having a maximum length of stay and the percentage of patients able to do very little without assistance: community based units scored 3.4 % lower than hospital units; rural units scored 3.3 % lower than those based in urban areas; larger units scored lower, every extra bed reducing the Therapeutic Environment domain score by 0.1 %; units with a maximum length of stay scored 9.0 % higher than those without; every 1 % increase in patients able to do very little without assistance was associated with a reduction in the Therapeutic Environment domain score of 0.1 %.

### Treatments and interventions

The Treatments and Interventions domain had an overall mean score across countries of 50.6 % (mean range 59.5 % [UK] to 46.2 % [Poland]). This was associated with whether the unit had a maximum length of stay and the percentage of patients able to do very little without assistance: those with a maximum length of stay scored 6.0 % more; for each 1 % increase in patients able to do very little without assistance, this domain score reduced by 0.1 %.

### Self-management and autonomy

The Self-Management and Autonomy domain had an overall mean score across countries of 55.5 % (mean range 71.9 % [Germany] to 44.1 % [Poland]). This was associated with the size of unit, whether it was single or mixed gender, the percentage of patients able to do very little without assistance and the percentage of patients detained: for every additional bed this domain score reduced by 0.2 %; single sex units scored 8.6 % lower than mixed gender units; for each 1 % increase in patients able to do very little without assistance, this domain score reduced by 0.2 %; for every 1 % increase in detained patients the score reduced by 0.1 %.

### Social interface

The Social Interface domain had an overall mean score across countries of 48.5 % (mean range 59.5 % [Spain] to 40.1 % [Poland]). This was associated with the type, size and location of the unit, having a maximum length of stay and the percentage of patients able to do very little without assistance. Community based units scored 7.8 % lower than hospital units; for every additional bed this domain score reduced by 0.2 %; units with a maximum length of stay scored 6.7 % higher than those without; and for every 1 % increase in patients able to do very little without assistance, this domain score reduced by 0.2 %.

### Human rights

The Human Rights domain score had an overall mean across countries of 56.7 % (range 70.8 % [Netherlands] to 48.1 % [Italy]). Scores were associated with whether the unit was mixed or single gender and the level of disability of the patients: single sex units scored 7.9 % lower than mixed gender units; and for every 1 % increase in patients able to do very little without assistance, this domain score reduced by 0.1 %.

### Recovery based practice

The Recovery Based Practice domain had an overall mean score across countries of 52.7 %, (mean range 65.9 % [UK] to 44.2 % [Portugal]). This was associated with the location of the unit, having a maximum length of stay, whether single or mixed gender, and the level of disability of the patients: units in rural areas scored 3.7 % lower than those based in urban areas; units with a maximum length of stay scored 5.8 % higher than those without; single gender units scored 4.8 % less than mixed gender units; and for every 1 % increase in patients able to do very little without assistance, this domain score reduced by 0.2 %.

## Discussion

### Main findings

Although there was wide variation in QuIRC domain scores between individual units, across countries, only 25 % of units scored less than 50 % on individual QuIRC domain scores. We found that a number of characteristics of units were associated with the quality of care delivered. The characteristic that had an influence on the largest number of domains was the level of disability of the patients; units with a higher proportion of poorer functioning patients scored lower on six of the seven QuIRC domains (all except Living Environment). This suggests that units that are managing a majority of people with high levels of disability may struggle to deliver better quality services and therefore having patients with a range of functioning may be important. However, since the number of units surveyed in most countries was relatively small (see below), we do not know whether this is an option as we do not know whether there is a range of disability of the patients in longer term units in different countries.

Whether the unit had a maximum length of stay was positively associated with four of the seven domains (Therapeutic Environment, Treatments and Interventions, Social Interface, and Recovery Based Practice). Having an expected length of stay is likely to help clarify the aims of treatment and instil a sense of hope in patients and staff. Units that focus on patients’ recovery and move on within a defined timeframe may make more effort to build links and informal support networks for patients in the local community and with their family and carers, and thus develop a more therapeutically optimistic environment. However, this variable is dependent on there being somewhere suitable for patients to move on to. It may be that a lack of appropriately supported community accommodation in many countries was one of the reasons why relatively few units had a maximum length of stay.

Larger units had poorer quality scores for four of the seven QuIRC domains (Living Environment, Therapeutic Environment, Self-Management and Autonomy, and Social Interface). This association might be explained in cases where units had previously been part of a large psychiatric hospital (prior to “deinstitutionalisation”) and perhaps continued the culture of the older institutions, promoting less autonomy of their patients. This finding also suggests that the risk of poorer quality, more institutional practice is greater in larger units and therefore the size of units is an important factor for policy makers and service planners to take into consideration when developing contemporary services that aim to promote individuals’ independence and community discharge.

Single sex units scored lower on three domains (Self-Management and Autonomy, Human Rights and Recovery Based Practice) which suggests that mixed gender units are preferable (wherever clinically appropriate).

The location of the unit did not seem to have a major influence on quality; however, those based in rural areas scored lower on the Therapeutic Environment and Recovery Based Practice domains, perhaps due to isolation and lack of access to other services.

The type of unit had the largest positive effect on any single domain, with community units scoring up to 11 % higher for “Living Environment” compared to hospital units. However, this characteristic was negatively associated with the Therapeutic Environment and Social Interface domain scores. These findings therefore only partially concur with previous studies that have reported that community based units provide a more homely and therapeutic environment that facilitates greater community access than hospital based facilities [4, 6].

We did not find that staffing intensity or staffing turnover were associated with any domain scores. However, we were unable to investigate the association of specific professions with the quality of care provided.

### Strengths and limitations

The sampling of units was not random and thus we cannot say that those surveyed are representative of the countries that took part. The units were invited to participate in a study to develop a quality indicator for assessing longer term residential mental health care, and though there were no eligibility criteria based on performance, it is possible that selection bias occurred. Units were known to the principal investigators, and may have represented above (or below) average quality for that country. Furthermore, given only 20 units participated in each country, we had insufficient power to make comparisons between countries. Although our results give a snapshot of provision for those with severe mental health problems requiring longer term care across Europe, it is important to stress that in some countries, such as Portugal, almost all relevant units were included in the sample, whereas in other countries, such as Germany, Italy and the UK, the sample comprised only a small proportion of units in one geographic area. A major strength of our study was the use of the QuIRC to assess quality. Not only has it been shown to be a reliable instrument [2], but it has excellent content validity since the individual items included were identified from triangulation of three different evidence sources. Thus it provides a standardised measurement of quality of care provided in the units studied, rather than a more subjective or value judgement. Our approach to the analysis was strengthened by taking account of clustering of units within a country and ensuring that the number of variables included in our regression analysis was appropriate for our sample size. Furthermore, we have previously found little positive association between service user characteristics and quality of care in these same longer term mental health facilities and in a national survey of longer term inpatient mental health units in England, so we are confident that the range of quality we found was not determined by service user variables [7, 9].

### Implications

The results of the study have important implications for policy and organisation of services. They show that the average quality of units for longer term patients in the ten European countries involved in the study, although not excellent, was quite reasonable: they scored more than 50 % in six of the seven domains and 48.5 % in the seventh domain. The results also show that only a quarter scored less than 50 % on individual QuIRC domain quality scores, including the Living Environment, Self-management and Autonomy, and Recovery Based Practice. This seems to suggest that better quality care is being provided than that which prevailed in European psychiatric institutions in the past, though of course we have no QuIRC data for the older institutions. However, across all domains there are still units with very low quality scores, indicating that a lot of work still remains to be done, both at the country and at the EU level.

Bearing these caveats in mind, our results are able to provide tentative guidance for those planning longer term mental health facilities. While this study cannot suggest an ideal size of unit, smaller units appear to have an advantage in terms of quality of care provision. Units situated in the community had considerably better quality scores for their living environment, and should, whenever possible, be provided rather than hospital based units. Having a larger proportion of patients with greater need was negatively associated with a number of quality domains and units should therefore avoid having only patients with severe disability or those who are detained, and instead try to work with people with a range of functioning. Finally, our results on gender would suggest that mixed sex units are to be preferred.

There are many possible socioeconomic reasons for the differences we found in domain scores across European countries. These include variability in the spend on mental health services; the history of service development; the level of service utilisation; the dispersal of settings for mental health care, including the criminal justice system; cultural norms about individualisation, self-management and family support; and the different expectations of the general public in different countries [10]. Ongoing collection of QuIRC data can provide an opportunity to collect and monitor national quality benchmarking for these services.

Two areas cannot be addressed from this study. Firstly, we do not know which of the QuIRC domains is *most* important in terms of patient experience of care and clinical outcomes. The identification of critical success factors for this group of patients would obviously help guide the organisation and management of services. Secondly, although the QuIRC provides an accessible and free tool that allows units to review their performance against national averages (www.quirc.eu), it is not yet known what interventions are feasible and effective in different contexts in helping staff to improve the quality of care they offer. Further study is required in both these areas.

## Conclusions

This is the first time longer term mental health units across a number of European countries have been compared using a standardised measure. The collection of QuIRC data constitutes the beginning of a database across Europe that will allow continuing assessment of quality of care in these units. It will also contribute to the development of realistic targets and timescales for the development of minimum quality of care standards for this stigmatised and vulnerable group of patients.
